# The Upworthy Research Archive, a time series of 32,487 experiments in U.S. media

**DOI:** 10.1038/s41597-021-00934-7

**Published:** 2021-08-02

**Authors:** J. Nathan Matias, Kevin Munger, Marianne Aubin Le Quere, Charles Ebersole

**Affiliations:** 1 Cornell University Department of Communication, Ithaca, USA; 2 Penn State University Department of Political Science, State College, USA; 3 Cornell University Department of Information Science, Ithaca, USA; 4 University of Virginia, Charlottesville, USA

**Keywords:** Human behaviour, Social sciences

## Abstract

The pursuit of audience attention online has led organizations to conduct thousands of behavioral experiments each year in media, politics, activism, and digital technology. One pioneer of A/B tests was Upworthy.com, a U.S. media publisher that conducted a randomized trial for every article they published. Each experiment tested variations in a headline and image “package,” recording how many randomly-assigned viewers selected each variation. While none of these tests were designed to answer scientific questions, scientists can advance knowledge by meta-analyzing and data-mining the tens of thousands of experiments Upworthy conducted. This archive records the stimuli and outcome for every A/B test fielded by Upworthy between January 24, 2013 and April 30, 2015. In total, the archive includes 32,487 experiments, 150,817 experiment arms, and 538,272,878 participant assignments. The open access dataset is organized to support exploratory and confirmatory research, as well as meta-scientific research on ways that scientists make use of the archive.

## Abstract (summary)

**Table:** 

Measurement(s)	behavior
Technology Type(s)	Web Site
Factor Type(s)	experimental group
Sample Characteristic - Organism	Homo sapiens
Sample Characteristic - Environment	internet

Machine-accessible metadata file describing the reported data: 10.6084/m9.figshare.14442737

## Background & Summary

Upworthy is a media publisher founded in 2012 to reach large audiences with “stuff that matters.” In pursuit of the largest possible audience, Upworthy conducted thousands of randomized behavioral experiments. These “A/B tests” estimated the rate at which viewers selected articles to read after viewing variations in “packages” of headlines and images. The dataset we present is a collection of every A/B test fielded by Upworthy between January 24, 2013 and April 30, 2015. The archive includes 32,487 experiments, 150,817 experiment arms, and 538,272,878 participant assignments. The archive also includes 78,232 packages that were not deployed or were not experiments.

Upworthy was a central actor in the history of U.S. media from 2013–2015. By the end of 2013, it was called the fastestgrowing media company in the world^1,2^, with its articles shared more frequently on Facebook than all U.S. mainstream media combined^3^. At the time, such experimentation was common in the technology industry and political campaigns, but novel in the context of online media. Upworthy’s content management infrastructure was designed to test every article first as a randomized trial, measure responses, and compare the probability of a viewer clicking on different potential packages for the same story. Editors would then choose the winning version for widespread publication.

As many publishers adopted headline styles similar to some of Upworthy’s most widely-spread stories, other media organizations complained about their success. In March of 2015, Upworthy co-founder Peter Koechley apologized for its out-sized success and pledged to change, saying ‘sorry we kind of broke the internet last year’^4^. As Upworthy changed its editorial practices, and other media organizations imitated their approach, social media platforms also changed their algorithms. In 2016, Facebook announced that it had adjusted the recommendation algorithms that ranked items on users’ News Feeds to reduce the visibility of content in styles attributed to Upworthy^5^. In 2017, Upworthy merged with Good Worldwide, the company which shared their historical data with us to create the Upworthy Research Archive^6^.

As a uniquely-large dataset of causal studies, The Upworthy Research Archive can be used to advance fields including communication, political science, psychology, media studies, computer science, marketing, and business. The stimuli being tested, the large number of experiments, and the context of Upworthy’s ascendancy in U.S. media make it a fruitful source for scholars across these disciplines. For each arm of an experiment, the Archive includes variables recording the date, number of clicks, number of impressions, and the text of the headlines; it does not include sales data, micro-level user data or advertising data. In political science and communication, the act of “clicking” is increasingly seen as a key but understudied component of the process by which an individual’s (political) media diet is constructed^7^. In psychology and marketing, scientists are interested in people’s preferential responses to stimuli that engage with reasoning and emotion. Computer scientists and statisticians formalize and create algorithms to process high volume flows of causal data and adapt in response. In business and organizational behavior, researchers study regimes of experimentation and how innovators use evidence to inform future decisions.

As a time-series of experiments, The Upworthy Research Archive can also support scientific investigation of temporal phenomena. The multi-year range of this dataset can help scholars understand the determinants of clicking behavior, whether these determinants are stable or contingent. With time-series information, researchers can also study changes in the behavior of an experimenting firm over time.

We hope this data can contribute to advances in quantitative methods as used by scientists and practitioners. Surveying the state of applied econometrics, Athey reports that the meta-analysis of many experiments is one of the most promising areas for future work^8^. With access to tens of thousands of behavioral experiments over time, statisticians can explore new avenues in meta-analytic statistical methodology.

The Upworthy Archive provides a unique glimpse into the high volume experimentation now being conducted by private firms. The authors of a recent summary article “are quite confident that there were more experiments on behavioral outcomes carried out by Facebook and Google this year than the sum total of those carried out by members of the American Political Science Associations Experimental Political Science section^9^. “So far, access to large corporate archives of experiments has been limited to a small number of scientists. As a result, much of the methodological research from large-scale corporate experimentation has been conducted by a small number of well-connected scholars (see, for example^10–12^). The dataset we describe here has the capacity to enable contributions from a wider range of scholars. We also expect this dataset to support advances in teaching about large-scale behavioral research, which has also been constrained by barriers to data access^13^.

As field experiments have become common in business, democracy, and the social sciences, experimenters have faced legitimate concerns about research ethics^14^. While academic researchers in the U.S. are required to follow the Common Rule, non-academic experimenters such as headline writers are not required to obtain committee review for news headlines or the design of their experiments^15^. Participant privacy is a also major risk in any scientific dataset involving people. Attempts to anonymize information can often be circumvented^16^. While sophisticated query algorithms can protect privacy while enabling scientific study^17^, it is much safer to avoid storing or sharing any individual-level data at all. The Cornell University IRB has determined that this project is not governed by U.S. research ethics regulations, since (a) the dataset does not include any information about individual people, and (b) the studies were conducted several years ago by Upworthy alone, with no involvement from us or our institutions.

## Methods

The experiments in this dataset randomly assigned readers of Upworthy.com to receive article previews and recorded whether each of them clicked on the preview or not. We refer to each article preview as an “experiment arm,” and the process of randomly determining which article preview each participant was shown as “assignment.” According to multiple sources, including the engineers who developed the website, Upworthy conducted only one experiment at a time on its website.

**Fig. 1:**
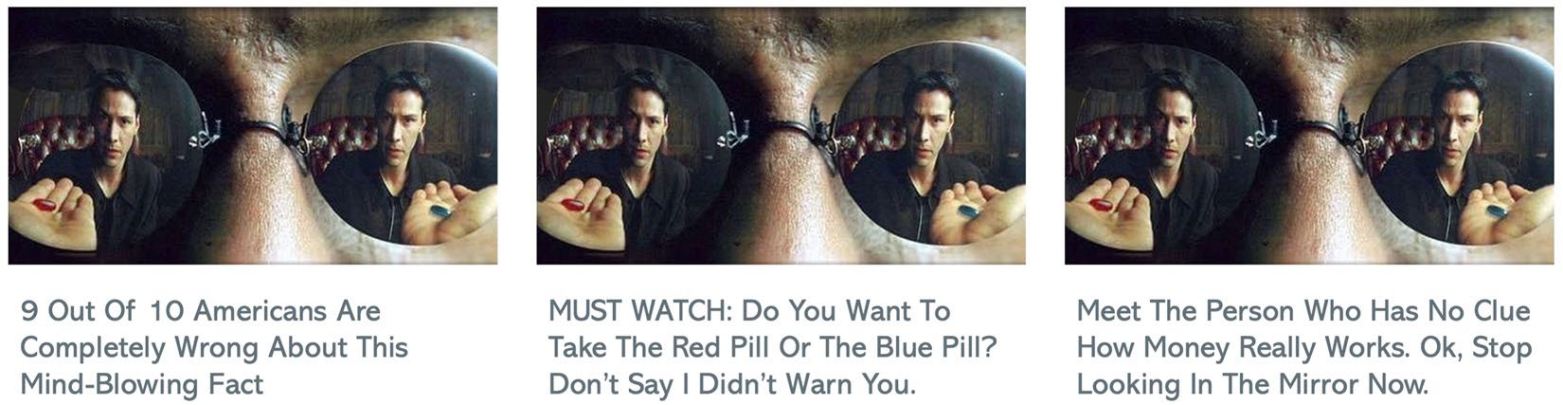
Reconstruction of one test in 2013 that was composed of different tested packages. For this particular test, 3 different headlines were compared and the image was kept constant.

For a single article on Upworthy, writers and editors created multiple variations of headlines, images, and in some cases descriptive text. These stimuli were bundled into a number of “packages” that constituted the arms of each experiment (Fig. 1). For example, a single experiment with two variations in images and two variations in headlines might have four arms, similar to a 2 × 2 experiment. Yet since Upworthy writers and editors exercised substantial judgment in the packages they created, few experiments explored all possible combinations. For example, an experiment that tests four headline variations might also test two image variations, but use only 4 arms rather than the 8 needed to test all possible combinations.

The unit of observation in this data is a web browser session accessing the Upworthy.com website. A participant was assigned to an experiment arm when their web browser requested a page from Upworthy. The website software, which was written in the Ruby Language (versions 2.9.3 through 2.3.1) used the RandomSample method^18^ to randomly assign the browser session to receive a given package, incorporating the package into the web page provided to the browser. The suspected exception to this randomization procedure took place between June 25, 2013 and January 10, 2014. During this period, we believe the website was verifiably randomizing experiment arms, but that the web cache that handled high volume requests was only serving one arm at a time. Upworthy only conducted one experiment at a time across the entire website, and pages never included more than one experiment. Packages were sometimes placed at the bottom of a page, outside the initial phone or desktop viewscreen and late in the reading order for audio screen-readers. Because the stimulus was not encountered to all participants, all experiments in this dataset should be considered “intent-to-treat” studies. In intent-to-treat studies, researchers estimate the average treatment effect of offering someone the treatment, whether or not they receive it^19^.

**Fig. 2:**
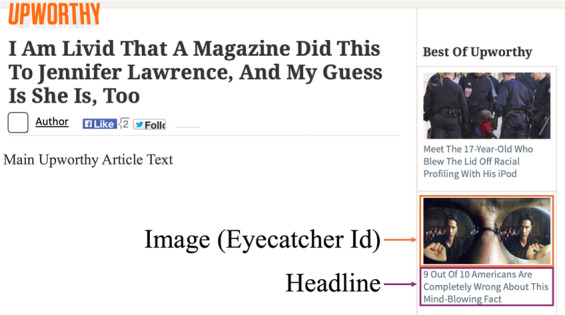
View of Upworthy website article view from December 2013. This figure demonstrates how the “Headline” and “Eyecatcher ID” fields from our dataset would have been shown to visitors. On this page, one of the image-headline combinations on the right sidebar was likely a package being tested.

The outcome variable for each experiment is whether a participant selected the package for further reading, whether that action was taken by clicking a computer mouse, tapping a touchscreen, or making the selection via other means. To guide researchers, the archive includes a series of time-stamped pictures of the website with information about where packages were placed as the website layout evolved (Fig. 2).

**Fig. 3:**
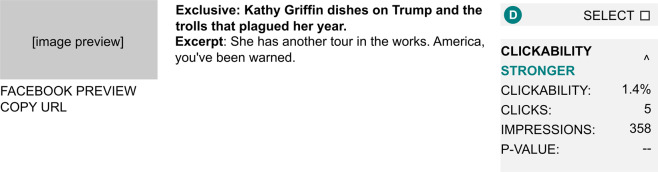
Editor view of packages in Upworthy’s testing system once testing was underway. This package (D), was the fourth arm in an experiment. This reproduction of the Farm software, from 2018, had an entry for p-value, which was not computed during the period covered by the archive.

Decisions to conclude an experiment were made by editors at Upworthy, who had access to a dashboard that reported the number of participants (“impressions”) and the number of clicks received by each arm of an experiment. They were also given a measure of “significance” for each arm, which was a custom calculation of a package’s relative success in the test (Fig. 3). Reviewing this information on an ongoing basis, editors would exercise judgment about when to halt the experiment. Editors would then decide if they should conduct a further test with new arms or choose which package to publish with an article across the Upworthy website. Editors sometimes exercised their judgment to choose a package other than the best performing one. From that decision onward, Upworthy would only display the final chosen package, creating space on website pages for another experiment.

## Data Records

The dataset is available under the Creative Commons Attribution 4.0 International License on the Upworthy Research Archive website at https://upworthy.natematias.com, with an archival copy on the Open Science Framework^20^. The website include a description of each column in the data, a list of resources and papers based on the dataset, and guidance for meta-analyzing the included experiments. The data are stored using a plain-text, ASCII-encoded, comma-delimited CSV file.

Each row in the dataset is a single package, or arm, in a given experiment. Since this is a historical archive, we have retained the column names used in the Upworthy software system (Table 1). Packages that were in the same experiment share the same *clickability_test_id*. The dataset includes 32,487 tests with an average of 5 packages per test.

**Table 1:** Columns in the Upworthy Research Archive.

Column name	Description
created_at	Time the package was created (timezone unknown)
test_week	Week the package was created, a variable constructed by the archive creators for stratified random sampling
clickability_test_id	The test ID. Viewers were randomly assigned to packages with the same test ID
impressions	The number of viewers who were assigned to this package. The total number of participants for a given test is the sum of impressions for all packages that share the same clickability_test_id
headline	The headline being tested
eyecatcher_id	Image ID. Image files are not available. Packages that shared the same image have the same eyecatcher_id
clicks	The number of viewers (impressions) that clicked on the package. The clickrate for a given package is the number of clicks divided by the number of impressions
excerpt	Article excerpt
lede	The opening sentence or paragraph of the story
slug	Internal name for the web address
share_text	Summary for display on social media when the article is shared. This was not shown in tests, since tests were conducted on the Upworthy website
square	When used, part of the same social media sharing suggestion as the share text
significance	NOT an estimate of statistical significance; a complex, inconsistent calculation that compared the clicks on a package to the clicks on all previous packages that were fielded on the same pages
first_place	Along with significance, shown to editors to guide decisions about what test to choose
winner	Whether a package was selected by editors to be used on the Upworthy site after the test
updated_at	The last time the package was updated in the Upworthy system
Problem	An indicator variable for whether the test took place during the problematic period

For each package, we record the time it was created (*created_at*) and the last time the package was updated in the Upworthy system (*updated_at*). From this information, we record a column with the week number that a package was created, starting with the earliest package in the dataset (*test_week*).

For each package, we record details of the stimulus presented to readers. The *headline* was presented to readers as a short, large-font text description of the article. The *eyecatcher_id* refers to an image that was also shown to readers as part of the package. Each package also includes an *excerpt* that was shown in a smaller font as part of the package. While this dataset does not include the images that were tested by Upworthy, packages within the same test that share the same image can be compared to each other. In the full dataset, 123,019 packages include an image that was included in at least one other package.

We also record several aggregate variables for each package. The *impressions* are the number of browser sessions that were randomly assigned to a given package during the experiment. The total number of participants in a given experiment is the sum of impressions across all packages in the experiment. The outcome variable is the number of *clicks* received by each package. Each participant could either select the package for further reading or not. Each participant is counted once; the record of clicks is the number of participants that selected the package. The dataset includes a total of 538,272,878 impressions and 8,182,674 clicks.

The dataset also includes information about the underlying article that was not shown to participants in A/B tests. The *lede* is the first sentence readers would see after selecting the article. The *slug* is an internal name for the package’s web address. The *share_text* was shown to participants after they selected an article, as a preview of the text that would be posted to their social media account if they shared the article with friends on social media. The *square*, when used, was part of the same social media sharing suggestion.

The dataset includes several columns used by editors in a system called the “Farm” that guided and recorded editorial decisions. The *significance* and *first_place* columns were shown to editors to guide decisions about what test to choose. Former staff report that the *winner* column identifies whether a package was selected by editors to be used on the Upworthy site after the test.

According a former lead engineer at Upworthy, the *significance* column reported the results of calculation that compared a given package’s click-through rate to packages in other, previous tests. This figure was generated by conducting, at intervals, a separate statistical test for every unique webpage on Upworthy that hosted the current experiment. The page-level click-through rate of each package was compared to the aggregate click-through rate for every other package that previously appeared in a test on the same page. The results of these thousands of tests were aggregated into a single, per-package measure of what the company called significance. The mathematical meaning of this field changed over time and is not consistent throughout the dataset. According to the former employee, “we updated this algorithm constantly to incorporate the most recent performance data as well as new findings and hypotheses.”

The *problem* column is a dummy variable we constructed when we became aware of the potential randomization problem for part of the dataset. This variable allows researchers to easily remove or include these tests.

In addition to fielded tests, we have also included all 78,232 non-fielded packages in a companion dataset in the archive. We also provide supplementary site-wide information about the Upworthy audience during the archive period. Upworthy used the Google Analytics service to collect and aggregate information about their audience. The Google Analytics audience data includes an aggregate country-level report from January 2013 through April 2015 of the number of viewers per country for 207 countries. Audience data also includes a daily report of the number of viewers, new viewers, average session duration, and page views for the same period.

## Technical Validation

The Upworthy Research Archive dataset is derived from internal software databases of Upworthy.com before the system was de-commissioned by the company. Because we did not personally conduct these studies or oversee the creation of this data, we have validated the research through a series of qualitative and quantitative methods. We collaborated with engineers, editors, and data scientists at the organization to document and validate the dataset. We also interviewed seven current and former employees to confirm details. Finally, we conducted quantitative checks of internal validity.

The dataset is a record of aggregated data from a system that Upworthy internally called the “Farm.” Editors used the system to review content, edit material, observe the results of tests, make decisions about ending a test, and choose the final form of an article. A variety of systems in the Upworthy infrastructure provided input to the Farm, which aggregated that information into ongoing results for the packages in a given test.

To validate the meaning of the fields involved, we interviewed current and former employees of Upworthy, including data scientists who created and maintained it and editors who used it on a regular basis. We showed them screenshots of the system, descriptions of the columns in the data, and screenshots of the Upworthy website. We asked interviewees to narrate their understanding of how the experiments were conducted, discussed the meaning of columns in the data, and asked them questions about statistical questions. Interviewees reviewed their email and software code archives to confirm the details.

We also conducted quantitative validation and filtering to create this final dataset. The Farm included 229,049 package records in total. Once we removed those that were not associated with a test and those that had never been fielded, the dataset included 150,817 packages. Non-fielded packages in the dataset included drafts that were never submitted for testing by writers, proposed packages that editors chose not to test, and test with only a single fielded package. Finally, we quantitatively validated fielded tests by confirming that clicks were always lower than the participant count and by checking the balance in the sample.

Subsequent analysis demonstrates conclusively that there was treatment imbalance for some of the tests^21^. The temporal distribution of these issues provides strong evidence that they only occurred between June 25, 2013 and January 10, 2014. The rest of the dataset is unaffected, but for the reasons discussed above, we conclude that the tests in this time period were not fully randomized.

Writers and editors describe testing as a process of imagining and comparing the most influential packages possible. First, they searched for online content that they saw as “awesome, meaningful, and visual,” that they believed could be more popular if they promoted it more effectively. They then created new articles on Upworthy that re-packaged this material for A/B testing and promotion. Writers and editors did not have a concept of a control group. Instead, they selected what they believed would be the most influential packages for testing. Upworthy also did not carry out power analysis. After fielding, editors would monitor a webpage with summary statistics similar to Fig. 3. When satisfied that enough information was collected, editors could halt the test and choose a final package. If editors were dissatisfied with the result, they could adjust the packages and start a new test. In the dataset, follow-up tests are recorded as a new test with new packages that have content in common with a previous test.

Upworthy staff believe that participant characteristics could vary substantially between tests due to selection processes common in web-based experiments. For example, if an article about a celebrity was widely shared and viewed on a given day, then people who tended to admire that celebrity would temporarily constitute the majority of test participants. If that article was no longer popular the next day, then an experiment the next day might have different demographics. Upworthy staff explained this belief by describing how readers arrived at articles where they encountered A/B tests. Readers often accessed Upworthy by following links on social media, culminating a sequence of selection processes that could lead to variation in participant characteristics between tests. This selection process was noticed by editors at moments when a single article was receiving disproportionately more readers than other articles, making that article’s readers a majority of experiment participants. Since many of Upworthy’s readers arrived on the site through social media, these participants were people whose social contacts had shared the article and who had chosen to follow the link. While we do not have quantitative evidence to confirm this belief, we report it as an important detail to consider when interpreting the experiments in the archive. Consequently, users of this dataset should not assume that two experiments close to each other in time have identical participant demographics or interests.

## Usage Notes

To support confirmatory research with the archive, we have also created three randomly sampled, exclusive subsets of tests. We stratified the randomization process to ensure that the three subsets were balanced in terms of the weeks the tests were conducted. The exploratory dataset includes 22,666 packages within 4,873 tests. The confirmatory dataset includes 105,551 packages within 22,743 tests. A hold-out dataset includes 22,600 packages within 4,871 tests. We developed these samples for use with a registered reports process of peer review.

When developing a study with data from the archive, we encourage researchers to be deliberate in making use of the datasets. Since this dataset reports findings from production software that might occasionally have experienced errors, we encourage researchers to pursue questions that incorporate many experiments across the exploratory and confirmatory datasets. For instance, researchers could conduct two rounds of exploratory analyses using the smaller datasets and then confirm their key findings in the much larger dataset (or use the exploratory results as the basis for a registered report). Conversely, the larger dataset could be used for high-powered exploration, and then the robustness of discoveries could be tested in the smaller datasets. If a researcher has strong a prioi hypotheses, they could simply utilize the full dataset in order to produce the most high-precision estimates of their hypotheses. Finally, those interested in meta-scientific research could give other researchers the datasets in stages to observe how hypotheses and analyses evolve with sequential testing. Overall, the data partitions provide several options for conducting multiple tests of research questions. We encourage researchers to use the datasets in an order that best supports their research goals (i.e., prioritizing exploration vs. confirmation).

We are making this dataset available under the Creative Commons Attribution 4.0 International License, which grants licensees the right to use, share, and adapt the dataset if they agree to attribute the archive and place no further restrictions on its use. We also make two non-binding requests. We ask that researchers contact the archive with information about publications so we can update a list of research that uses it. We also request that researchers openly publish the analysis code for their studies and contribute that code to the Upworthy Research Archive.

## Data Availability

The code for creating computationally-reproducible research samples is available as part of the archive.
