# Author Correction: The Upworthy Research Archive, a time series of 32,487 experiments in U.S. media

**DOI:** 10.1038/s41597-024-03600-w

**Published:** 2024-07-11

**Authors:** J. Nathan Matias, Kevin Munger, Marianne Aubin Le Quere, Charles Ebersole

**Affiliations:** 1 Cornell University Department of Communication, Ithaca, USA; 2 Penn State University Department of Political Science, State College, USA; 3 Cornell University Department of Information Science, Ithaca, USA; 4 University of Virginia, Charlottesville, USA

Correction to: *Scientific Data* 10.1038/s41597-021-00934-7, published online 02 August 2021

Following publication, we (the authors) discovered evidence of likely problems with the randomization of the tests between June 25, 2013 and January 10, 2014. A total of 7,004 A/B tests or 22% of experiments may have been affected. Based on further interviews with engineers and follow-up analysis (see the supplementary file associated with this correction notice), we believe that the website likely had a caching issue that prevented headlines from being randomly assigned to viewers. During this period, we observed an imbalance in the number of impressions received by arms in A/B tests. While configuration details from the third-party caching service were unavailable, we and former Upworthy engineers hypothesize that although the website was verifiably randomizing experiment arms, the web cache that handled high volume requests was only serving one arm at a time. We have found timing patterns in the degree of arm imbalance that are consistent with a configuration error of this kind (Fabijan *et al*. 2019). Consequently, we encourage researchers to treat these tests as not randomized.

In response to this discovery, we encourage researchers conducting causal analysis to omit all experiments from June 25, 2013 through the end of January 10, 2014.

We have attached our full analysis of the randomization imbalance issue as a supplementary file and made the following updates to the paper and dataset to support this:

New text added to the Methods section:

“The suspected exception to this randomization procedure took place between June 25, 2013 and January 10, 2014. During this period, we believe the website was verifiably randomizing experiment arms, but that the web cache that handled high volume requests was only serving one arm at a time.”

An addition variable called “problem” added to the main datafile. The name definition of this column heading has been added to table 1 and the following text added to the Data Record section:

“The problem column is a dummy variable we constructed when we became aware of the potential randomization problem for part of the dataset. This variable allows researchers to easily remove or include these tests.”

Text added to the Technical Validation section:

“Subsequent analysis demonstrates conclusively that there was treatment imbalance for some of the tests. The temporal distribution of these issues provides strong evidence that they only occurred between June 25, 2013 and January 10, 2014. The rest of the dataset is unaffected, but for the reasons discussed above, we conclude that the tests in this time period were not fully randomized.”

A reference has been added to the reference list:

Fabijan, A. *et al*. Diagnosing sample ratio mismatch in online controlled experiments: a taxonomy and rules of thumb for practitioners. In *Proceedings of the 25th ACM SIGKDD International Conference on Knowledge Discovery & Data Mining* (pp. 2156-2164) (July, 2019).

We are very grateful to Dean Eckles and Garrett Johnson for alerting us to the issue of randomization imbalance and for input on diagnosing it. This acknowledgement has also been added to the original paper.

